# Association between Body Mass Index and Immune-Related Adverse Events (irAEs) among Advanced-Stage Cancer Patients Receiving Immune Checkpoint Inhibitors: A Pan-Cancer Analysis

**DOI:** 10.3390/cancers13236109

**Published:** 2021-12-03

**Authors:** Dongyu Zhang, Neil J. Shah, Michael Cook, Matthew Blackburn, Michael T. Serzan, Shailesh Advani, Arnold L. Potosky, Subha Madhavan, Anas Belouali, Michael B. Atkins, Dejana Braithwaite

**Affiliations:** 1 Department of Epidemiology, College of Public Health and Health Professions, University of Florida, Gainesville, FL 32610, USA; dzhang2@ufl.edu; 2 University of Florida Health Cancer Center, Gainesville, FL 32610, USA; 3 Department of Medicine, Solid Tumor Genitourinary Oncology Service, Memorial Sloan Kettering Cancer Center, New York, NY 10065, USA; shahn6@mskcc.org; 4 Department of Medicine, Division of Hematology/Oncology MedStar Georgetown University Hospital, Washington, DC 20007, USA; michael.r.cook@gunet.georgetown.edu (M.C.); matthew.j.blackburn@gunet.georgetown.edu (M.B.); michael.t.serzan@gunet.georgetown.edu (M.T.S.); 5 Department of Oncology, School of Medicine, Georgetown University, Washington, DC 20007, USA; sa1542@georgetown.edu; 6 Transplant Education Research Center, Terasaki Institute of Biomedical Innovation, Los Angeles, CA 90024, USA; 7 Georgetown Lombardi Comprehensive Cancer Center, Washington, DC 20057, USA; arnold.potosky@georgetown.edu (A.L.P.); sm696@georgetown.edu (S.M.); anas.belouali@georgetown.edu (A.B.); 8 Innovation Center for Biomedical Informatics, Georgetown University Medical Center, Washington, DC 20007, USA; 9 Department of Surgery, University of Florida, Gainesville, FL 32610, USA

**Keywords:** immune checkpoint inhibitor, body mass index, immune-related adverse events, epidemiology

## Abstract

**Simple Summary:**

Currently, clinical studies exploring the impact of high body fat on toxicities after receiving immune checkpoint inhibitors (ICIs) among cancer patients are limited. Here, we analyze data from a health care system serving the mid-Atlantic geographic region to assess how body fat can affect the development of toxicities of ICIs. In our study, body mass index (BMI) was used as the measure of body fat, and the results suggested that cancer patients with a high BMI were more likely to have toxicities after receiving ICIs. Our study suggests that symptom management should be incorporated in the cancer care continuum of patients who receive ICIs, especially those with high BMI. In clinical settings, oncologists should inform cancer patients receiving ICIs with high BMI that their risk of post-treatment toxicities can be higher compared to their counterparts with lower BMI.

**Abstract:**

Evidence regarding the association between body mass index (BMI) and immune-related adverse events (irAEs) among cancer patients receiving immune checkpoint inhibitors (ICIs) is limited. Here, we use cross-sectional hospital-based data to explore their relationship. Pre-treatment BMI was treated as an ordinal variable (<25, 25 to ≤30, ≥30 kg/m^2^). The outcome of interest was irAEs after ICI initiation. A multivariable logistic regression model estimated the adjusted odds ratio (aOR) and 95% confidence interval (CI) of BMI. A total of 684 patients with stage III or IV cancer were included in the study (lung: 269, melanoma: 204, other: 211). The mean age at the first dose of ICI was 64.1 years (SD = 13.5), 394 patients (57.6%) were male, and over one-third (*N* = 260, 38.0%) were non-White. Overall, 52.9% of patients had BMI ≥ 25 kg/m^2^ (25 to ≤30: 217, ≥30: 145) and 288 (42.1%) had irAEs after ICI treatment. Patients with higher BMI tended to have a higher rate of irAEs (<25: 35.7%, 25 to ≤30: 47.0%, ≥30: 49.0%). The multivariable logistic regression yielded consistent results (BMI ≥ 30 vs. BMI < 25: aOR = 1.47, 95% CI = 0.96–2.23; 25 ≤ BMI < 30 vs. BMI < 25: aOR = 1.46, 95% CI = 1.02–2.11, *p*-trend = 0.04). In conclusion, among patients with advanced cancer receiving ICIs, the rate of irAEs appears to be higher among those with higher BMI.

## 1. Introduction

Immune checkpoint inhibitors (ICIs), such as anti-cytotoxic T lymphocyte-associated antigen 4 (anti-CTLA-4), anti-programmed cell death 1 (anti-PD-1), and anti-programmed death-ligand 1 (anti-PD-L1), have revolutionized the management of malignant tumors, especially those at advanced stages [1,2,3]. By unleashing immune activity, ICIs may facilitate cancer regression and improve survival when administered as either first-line therapy or after other treatment modalities have failed [4,5,6]. However, as indications for ICI therapy have expanded to treat increasing numbers of patients, there are growing concerns regarding immune-related adverse events (irAEs), including potentially severe adverse events affecting major organ function and/or quality of life [7,8,9,10]. Some fatal outcomes related to irAEs have been reported as well [11,12]. In 2018, the American Society of Clinical Oncology and the National Comprehensive Cancer Network published guidelines regarding the management of adverse events associated with immunotherapy [13,14].

Body mass index (BMI)—an index that can be easily measured in clinical settings—has been widely used to assess body fat in human subjects [15]. Clinical evidence suggests that high body fat can increase the likelihood of developing treatment toxicities in cancer patients receiving systemic treatment by affecting multiple signaling pathways (e.g., inflammatory, metabolic) [16]. Thus, improving our understanding of the impact of BMI on the safety of ICIs is important for clinical practice, particularly in terms of identifying patient subgroups that may require additional monitoring during therapy. Notably, extant studies evaluating the relationship between BMI and irAEs in patients with cancer receiving ICIs are limited due to the relative novelty of this treatment modality versus other cancer therapies that have been used for decades. In addition, previous studies of associations between BMI and irAEs in patients receiving ICIs have methodological limitations [17,18,19], such as limited racial/ethnic diversity of the study population and small sample sizes with potentially large random errors, possibly compromising their robustness.

To elucidate the association between BMI and irAEs induced by ICIs and provide relevant evidence in the management of patients with advanced-stage cancer, we conducted a cross-sectional analysis of diverse patients with advanced-stage cancer treated within a health care system serving the mid-Atlantic geographic region.

## 2. Methods

### 2.1. Immuno-Oncology Database and Study Population

**Figure 1:**
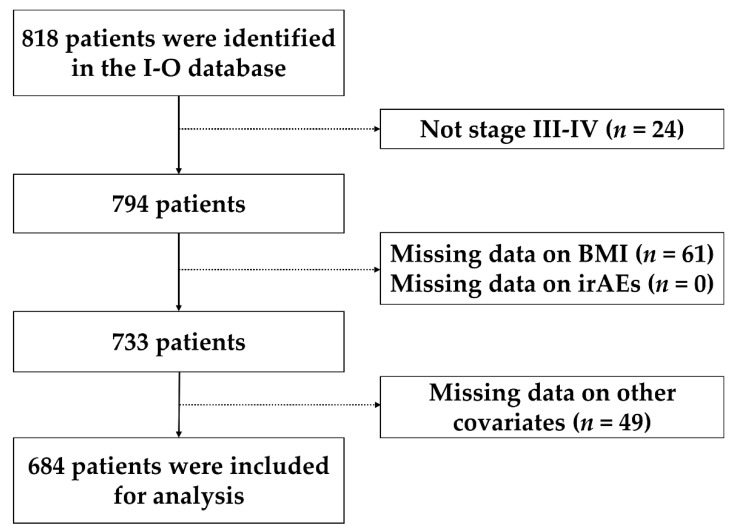
Flowchart for study participants selection. Abbreviations: BMI: body mass index, irAEs: immune-related adverse events.

We used a centralized research data warehouse for immuno-oncology (I-O) database built for real-world data analysis over a 7-year period. The comprehensive I-O database at MedStar Health Hospitals was developed to track and assess all patients with advanced cancer treated with ICIs. Between January 2011 and April 2018, information was collected retrospectively on patients treated with ICIs at five MedStar Health hospitals (two DC area hospitals: MedStar Georgetown University Hospital, MedStar Washington Hospital Center; and three hospitals at Baltimore: MedStar Franklin Square Hospital, MedStar Good Samaritan Hospital, and MedStar Union Memorial Hospital). A total of 818 subjects treated with ICIs were identified in the I-O database, and they were included in the analysis if they had the following characteristics: (1) had stage III or IV cancer, (2) had pre-treatment BMI, (3) information on irAEs was not missing, and (4) had no missing values of other study covariates. This yielded a total of 684 (83.6%) participants for analysis (Figure 1).

### 2.2. Measures

The exposure of interest in our study was pre-treatment BMI. Specifically, patients’ pre-treatment height and body weight were extracted using SQL queries from electronic health records. BMI was calculated as body weight (kg) divided by height (m) squared and categorized as an ordinal variable (BMI < 25, 25 ≤ BMI < 30, and BMI ≥ 30) based on cutoffs suggested by the World Health Organization [20]. The outcome of interest was irAEs, which were verified for each patient by the investigators using Common Terminology Criteria for Adverse Events (CTCAE) v 4.03. The I-O database measures the following 15 adverse health events: colitis, hepatitis, skin rash, pruritus, other skin toxicities, pneumonitis, hypothyroidism, hyperthyroidism, hypophysitis, other endocrine toxicities, immune-related joint pain/arthritis, immune-related neurological toxicity, immune-related hematological toxicity, musculoskeletal toxicity, and other toxicities. For the current analysis, we treated irAEs as a binary variable (no irAEs vs. had any irAEs). Pharmacy records were used to identify the history of ICI receipt, which included detailed information regarding the utilization of anti-PD-1 pathway blockers (nivolumab, pembrolizumab, atezolizumab, durvalumab, and avelumab), anti-CTLA-4 (ipilimumab), and combined modalities (ipilimumab plus nivolumab). Other relevant covariates regarding disease, patients’ demographic characteristics, and health-related behaviors were extracted from the electronic health records using SQL queries as well. Age at the first dose of ICI, sex, race, and smoking status at ICI initiation (never, current, former smoker) were obtained from patients’ self-reported information in health records. A total of 34 types of pre-existing comorbidities, which incorporated cardiovascular diseases, metabolic disorders, kidney disease, psychological disorders, prior cancer history, and infectious diseases, were considered for this current study. Patients’ functional status in terms of their ability to care for themselves, daily activity, and physical ability was reflected by their pre-treatment Eastern Cooperative Oncology Group (ECOG) Performance Status (PS); this index ranges from 0 to 5, with 0 suggesting no functional impairment and 5 indicating death [21].

### 2.3. Statistical Analysis

In the analysis, we first reported the number of observations and distribution of study covariates in the overall sample and by the number of irAEs (0, 1, and ≥2). A chi-squared test was used to examine if the distribution of study covariates differed by the number of irAEs; if the number of observations in any cell was smaller than 5, Fisher’s exact test was used. A descriptive summary of distribution of each individual type of irAE was conducted as well. The number of patients with irAEs and the rate of irAEs were summarized according to pre-treatment BMI categories, and we calculated point estimates as well as the 95% confidence interval of the irAE rate by BMI categories. An unadjusted and multivariable logistic regression model was used to estimate crude and adjusted odds ratios (ORs) for BMI, respectively; in these models, BMI < 25 kg/m^2^ was treated as the reference. The multivariable model adjusted for age (≤54, 55–64, 65–74, and ≥75 years), race (White, Black, and other), sex (male and female), smoking status (never, former, and current smoker), metastasis (yes vs. no), and lines of therapy (1 vs. 2+). The selection of confounders was based on *a priori* knowledge regarding the impacts of these covariates on BMI and treatment toxicities [22,23,24,25,26]. Specifically, smoking status was treated as an indicator of lifestyle behaviors that can affect BMI. Patients with metastatic cancer were more likely to have frailty that could affect body composition and risk of treatment toxicity [27], and patients receiving ICIs as the second (or more) line of therapy could have a differential physiological profile induced by prior therapies, which impacts metabolic homeostasis [28], suggesting that metastasis and line of therapy might be confounders between BMI and irAEs. A test for trend was conducted by treating BMI as a continuous variable in the model. As to the dose–response relationship between BMI and irAEs, we used a restricted cubic spline in the multivariable logistic regression model; specifically, we treated BMI = 21.8 kg/m^2^ as the reference in the dose–response curve because it was the mean value among patients with BMIs lower than 25 kg/m^2^. We assessed the non-linearity of the dose–response curve by comparing the model fit of the restricted cubic spline with a model fit assuming linearity for BMI via a likelihood ratio test [29]. A histogram was depicted along with the dose–response curve to summarize the distribution of pre-treatment BMI in our study population. We applied multiple imputations fit with 5 replicates of chained equations to assess if missing data impacted the association pattern of BMI. Subgroup analyses were conducted by age (<65 vs. ≥65 years), sex (male vs. female), race (White vs. non-White), burden of comorbidity (no multimorbidity vs. had multimorbidity), pre-treatment ECOG PS (<2 vs. ≥2), cancer type (lung vs. melanoma), dose of ICI (1–4 vs. ≥5), and ICI (nivolumab, pembrolizumab, and ipilumumab) using the same multivariable model. We chose multimorbidity and ECOG PS as an indicator for stratification because previous studies suggested that a higher burden of co-existing illnesses was associated with chronic inflammation [30,31] which could be a mediator between BMI and irAEs. We specifically compared association patterns between different cancer types, ICIs, and dose due to the potential biological heterogeneity across them. In subgroup analyses, BMI was treated as a binary variable (BMI ≥ 25 vs. BMI < 25) for sample size consideration, and we tested for interaction effects between BMI and the aforementioned factors used for subgroup analyses via a Wald test. In sensitivity analysis, we applied a multivariable linear regression model to investigate association between pre-treatment BMI and number of irAEs; in this linear regression, we adjusted for the same set of covariates as the primary logistic regression and estimated adjusted mean difference (aMD) and 95% CI for pre-treatment BMI. Two-sided *p*-values <0.05 were considered to be statistically significant. All statistical analyses were conducted in Stata version 15.0 (StataCorp, LLP, College Station, TX, USA).

## 3. Results

Table 1 presents the characteristics of our study population by number of irAEs. A total of 288 (42.1%) participants had at least 1 irAE (164 had 1 and 124 had ≥2). The study population had a mean age at ICI initiation of 64.1 years (SD = 13.5); 57.6% of them were male, and 62.0% and 26.6% self-reported as White or Black, respectively. Over half of patients had a smoking history (former: 48.3%, current: 9.6%), 12.0% were living without comorbidities, and over three-fourths (78.5%) of patients had ECOG PS < 2. Among these patients, 39.3% and 29.8% had lung cancer or melanoma, respectively, and 82.5% had documented metastases. Over one-third of patients (38.3%) received ICI as their first-line therapy. Over half (54.4%) of patients received less than 5 doses of ICIs. Nivolumab (38.5%), pembrolizumab (27.9%), and ipilimumab (11.8%) were the most commonly used ICIs, and about ten percent (10.4%) of patients used nivolumab plus ipilumumab. Patients with a higher number of irAEs were more likely to be White, have fewer baseline comorbidities or lower ECOG PS scores, have melanoma, use ICI as the first-line therapy, receive a higher number of doses of ICIs, and have non-pembrolizumab ICIs (*ps* < 0.05). Rates of colitis (10.1%), hepatitis (9.8%), skin rash (16.4%), pruritus (5.0%), and hypothyroidism (7.9%) were relatively higher compared to other irAEs (Table S1).

The mean BMI was 26.1 kg/m^2^ (SD = 5.9); over half (52.9%) of the patients had BMI ≥ 25 kg/m^2^ (25 to ≤30: 217, ≥30: 145). The rate of irAEs became higher as BMI increased, although the point estimates were similar between overweight and obese patients (BMI < 25: 35.7%, 25 ≤ BMI < 30: 47.0%, BMI ≥ 30: 49.0%). The unadjusted model suggested positive associations between BMI and irAEs, and this pattern did not change in the multivariable model (BMI ≥ 30 vs. BMI < 25: aOR = 1.47, 95% CI = 0.96–2.23, 25 ≤ BMI < 30 vs. BMI < 25: aOR = 1.46, 95% CI = 1.02–2.11, *p*-trend = 0.04) (Table 2). Effect measures of other covariates in the multivariable logistic regression model are present in Table S2.

**Figure 2:**
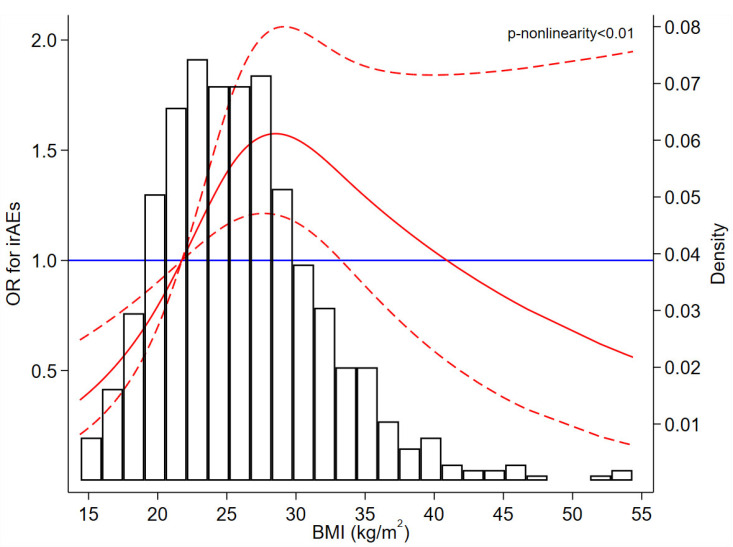
Plot depicting the distribution of pre-treatment BMI and the dose–response relationship between pre-treatment BMI and irAEs. BMI = 21.8 was treated as a reference in the dose-response curve. The solid red line is the fitted line, red dash lines are 95% confidence intervals of OR, and the blue solid line is the reference line. Abbreviations: BMI: body mass index, irAEs: immune-related adverse events, OR: odds ratio.

The dose–response curve showed a non-linear relationship between BMI and irAEs, and the curve was statistically significant when BMI was lower than approximately 34 kg/m^2^; specifically, the odds of irAEs reached the highest value when BMI was about 29 kg/m^2^ (Figure 2). The histogram shows that only a small fraction of participants had extremely high pre-treatment BMI (Figure 2). For example, only 47 (6.9%) patients had pre-treatment BMI ≥ 35 kg/m^2^ and 17 (2.5%) had pre-treatment BMI ≥ 40 kg/m^2^. The results obtained after multiple imputations were largely unchanged (Table S3).

Results of subgroup analyses are presented in Table 3. Significant interactions were identified for age and multimorbidity. The association between overweight (BMI ≥ 25 vs. BMI < 25) and irAEs was positively significant for patients younger than 65 years, whereas the association for older participants (≥65 years) was almost null (<65: aOR = 2.18, 95% CI = 1.36–3.51, ≥65: aOR = 1.08, 95% CI = 0.69–1.69, *p*-interaction = 0.02). Among patients without multimorbidity, people with higher BMI had a 3.20-fold relative increase in odds of having irAEs (BMI ≥ 25 vs. BMI < 25: aOR = 4.20, 95% CI = 2.11–8.37), but the association was null for those with multimorbidity (BMI ≥ 25 vs. BMI < 25: aOR = 1.03, 95% CI = 0.67–1.52, *p*-interaction < 0.01). In addition, the Wald test did not suggest a significant interaction between ICI type and overweight in relation to irAEs (for nivolumab: aOR_[BMI ≥ 25 vs. BMI < 25]_ = 0.93, 95% CI = 0.54–1.60; for pembrolizumab: aOR_[BMI ≥ 25 vs. BMI < 25]_ = 1.92, 95% CI = 1.00–3.71; for ipilumumab: aOR_[BMI ≥ 25 vs. BMI < 25]_ = 1.21, 95% CI = 0.55–2.65; *p*-interaction = 0.21), although the point estimates were largely different.

Although point estimates obtained from multivariable linear regression were non-significant (Table S4), the *p*-trend (0.045) suggested statistical significance and indicated a potential positive dose–response relationship between BMI and the severity of irAEs.

## 4. Discussion

Overall, our research suggests that about 40% of patients with advanced-stage cancer receiving ICIs had irAEs. In the multivariable model, patients with a higher BMI at ICI initiation had a higher rate of irAEs compared to patients with BMI under 25 kg/m^2^. Although the dose–response curve becomes non-significant when BMI is higher than about 34 kg/m^2^, the significant part of the curve suggests that patients who are overweight or obese are at higher odds of irAEs than those with lower BMI. We speculate that the wide 95% CI and inverse association observed in the right tail of the dose–response curve are caused by sparse data of observations with high BMI. The subgroup analyses of age and multimorbidity indicate that the impact of BMI on irAEs is much stronger among younger and healthier patients. One potential reason is that people with younger age and lower burden of comorbidities have more favorable homeostasis and are less likely to have immunosenescence [32,33]; this suggests that younger and healthier patients tend to have a higher likelihood of treatment-induced immune reactivity, which can be an upstream event of irAEs [34,35]. Thus, high BMI [36,37] synergistically interacts with treatment-induced immunity and increases the likelihood of irAEs among these younger and healthier patients with cancer, whereas these synergistic effects are not observed in older and more vulnerable patients because they may not have a strong response to ICIs due to immune senescence. The impact of BMI appears to be more substantial in patients receiving pembrolizumab than nivolumab and ipilumumab, although there is no significant interaction. However, the distributions of cancer type for each individual ICI treatment modality were quite different; thus, we cannot determine if the magnitude of interaction between BMI and ICI is affected by the heterogeneity of cancer type.

To our knowledge, there are two meta-analyses exploring the association between BMI and irAEs among patients with cancer receiving ICIs, and a total of 10 effect measures of BMI from published studies or abstracts were included for quantitative synthesis in these meta-analyses [38,39]. Although both of them concluded that high BMI was associated with a higher rate of irAEs, methodological limitations in the meta-analyses and included studies should be considered. Particularly, only 3 [17,18,19] of these effect measures included for synthesis were from published original studies that adjusted for potential confounders, indicating that the results of these meta-analyses could be biased to some extent. Among the studies included in the aforementioned meta-analyses, Daly et al. analyzed 84 patients with stage IV melanoma treated with ipilimumab and found that overweight (BMI ≥ 25 kg/m^2^) was associated with a 3-fold increase in odds of irAEs (OR = 4.01, 95% CI = 1.03–15.69) [18]. Another study conducted in France investigated 92 patients with stage IV cancer (lung, kidney, or melanoma) and found a positive association between BMI and irAEs (BMI ≥ 25 vs. <25: OR = 5.94, 95% CI = 1.25–28.29) [19]. However, the wide 95% CIs in these studies indicate that statistical imprecision may be induced by their small sample sizes. Our study used a larger sample size and investigated the relationship in different subgroups; this induces less random error and provides evidence for oncologists so that they can better identify patients whose risk of irAEs is more likely to be affected by high BMI.

The underlying mechanisms regarding the effects of BMI on irAEs remain to be determined. One speculation is that pharmacokinetic changes induced by obesity can impact the absorption, distribution, metabolism, and excretion of ICIs, and such change has the potential to affect the risk of irAEs following the utilization of ICIs [18]. Another explanation is that the ICI dosage was based on patients’ weight before 2018 [40]; thus, our patients with higher BMI (who were mainly treated before 2018) were more likely to have received a higher dose of ICI, which could ultimately increase the likelihood of irAEs. However, a flat dose has been widely used in ICI treatment modalities after 2018, indicating that the “BMI-dose–irAEs” relationship may be diminished to some extent for cancer patients receiving ICIs currently.

Our study has several methodological strengths. Health information was obtained directly from medical records, which is more valid than self-reports. The large sample size of our dataset ensured good power and precision in effect estimates. As compared to previously published studies investigating a similar topic, we examined the interaction between BMI and other covariates in relation to irAEs, which allowed us to explore factors inducing heterogeneity in the association between BMI and irAEs in cancer patients. The application of restricted cubic spline allowed us to assess the impact of BMI on irAEs from a non-linear dose–response perspective. However, several limitations should be noted when interpreting our results. First, this is a cross-sectional analysis without follow-up; thus, we are unable to conduct a time-to-event analysis to assess how soon these irAEs occurred. Second, to ensure better statistical power, we pooled cases of all types of cancer in the sample, which might have introduced some clinical heterogeneity because the underlying pathogenesis characteristics of various types of cancer can be different. Similarly, we summed all types of irAEs in logistic regressions since the number of each individual irAE was small. Although all of these study participants received ICIs, the specific type of ICI can be different. In clinical practice, nivolumab is more likely to be used for lung cancer, whereas ipilimumab is usually prescribed for melanoma; furthermore, 152 patients in our study received ipilumumab, but about half (*n* = 71) of them received nivolumab simultaneously, which made it hard for us to explore the effects of BMI on toxicities induced by each type of ICI among this subgroup. All these heterogeneities suggest that future studies with larger samples of irAEs and more homogeneous populations are needed to further disentangle the relationship between BMI and irAEs. Lastly, since both ICI utilization and the pre-existing burden of comorbidities can contribute to the development of adverse events in our study population, we could not determine if these adverse events were caused by ICIs or the synergistic effects between ICIs and comorbidities.

Our study has some health implications. In clinical settings, patients with advanced-stage cancer and a high BMI should be informed of the potential higher likelihood of irAEs before receiving ICI treatment. Oncologists should inform cancer patients with high BMI that their risk of irAEs can be higher compared to their counterparts with lower BMI. Symptom management should be incorporated in the cancer care continuum of patients who receive ICIs for treatment, especially those with high BMI. Currently, some ongoing randomized controlled trials (RCTs) are investigating interventional strategies that can reduce the risk of irAEs in cancer patients receiving ICIs as well as the underlying biological mechanisms. However, to date, no completed RCTs regarding irAEs management have been published [41,42]. Our results may provide a new target for intervention in future RCTs aimed at controlling irAEs in cancer patients.

## 5. Conclusions

In conclusion, our study suggests that higher pre-treatment BMI is associated with a higher rate of irAEs among patients with advanced-stage cancer receiving ICIs, especially those with younger age or a low burden of comorbidities. Future prospective cohort studies with clear temporality will be needed to verify our results from a causal perspective.

## Figures and Tables

**Table 1:** Summary of study characteristics.

Variables	Overall (*N* = 684)	irAEs			*p*-Value *
		0 (*N* = 396)	1 (*N* = 164)	≥2 (*N* = 124)	
	*N* (%)	*N* (%)	*N* (%)	*N* (%)	
Age at first dose (years)					
≤54	147 (21.5)	77 (19.4)	36 (21.9)	34 (27.4)	0.50
55–64	175 (25.6)	98 (24.8)	44 (26.8)	33 (26.6)	
65–74	207 (30.3)	124 (31.3)	48 (29.3)	35 (28.2)	
≥75	155 (22.7)	97 (24.5)	36 (21.9)	22 (17.7)	
Sex					
Male	394 (57.6)	224 (56.6)	88 (53.7)	82 (66.1)	0.09
Female	290 (42.4)	172 (43.4)	76 (46.3)	42 (33.9)	
Race					
White	424 (62.0)	220 (55.6)	107 (65.2)	97 (78.2)	<0.01
Black	182 (26.6)	130 (32.8)	39 (23.8)	13 (10.5)	
Other	78 (11.4)	46 (11.6)	18 (11.0)	14 (11.3)	
Smoking status					
Never	288 (42.1)	156 (39.4)	69 (42.1)	63 (50.8)	0.27
Former	330 (48.3)	201 (50.8)	78 (47.6)	51 (41.1)	
Current	66 (9.6)	39 (9.8)	17 (10.4)	10 (8.1)	
Comorbidities					
0	82 (12.0)	40 (10.1)	18 (11.0)	24 (19.4)	0.04
1	131 (19.2)	80 (20.2)	30 (18.3)	21 (16.9)	
2	157 (22.9)	88 (22.2)	34 (20.7)	35 (28.2)	
≥3	314 (45.9)	188 (47.5)	82 (50.0)	44 (35.5)	
ECOG PS at first dose					
0	189 (27.6)	75 (18.9)	52 (31.7)	62 (50.0)	<0.01
1	348 (50.9)	203 (51.3)	87 (53.1)	58 (46.8)	
≥2	147 (21.5)	118 (29.8)	25 (15.2)	4 (3.2)	
Cancer type					
Lung	269 (39.3)	184 (46.5)	59 (36.0)	26 (21.0)	<0.01
Melanoma	204 (29.8)	75 (18.9)	53 (32.3)	76 (61.3)	
Other	211 (30.9)	137 (34.6)	52 (31.7)	22 (17.7)	
Metastasis					
No	120 (17.5)	62 (15.7)	35 (21.3)	23 (18.6)	0.26
Yes	564 (82.5)	334 (84.3)	129 (78.7)	101 (81.4)	
Lines of ICI therapy					
1	262 (38.3)	126 (31.8)	63 (38.4)	73 (58.9)	<0.01
2	290 (42.4)	177 (44.7)	73 (44.5)	40 (32.3)	
≥3	132 (19.3)	93 (23.5)	28 (17.1)	11 (8.9)	
ICI dose					
1–2	186 (27.2)	140 (35.4)	29 (17.7)	17 (13.7)	<0.01
3–4	186 (27.2)	98 (24.7)	52 (31.7)	36 (29.0)	
5–10	156 (22.8)	83 (21.0)	45 (27.4)	28 (22.6)	
≥11	156 (22.8)	75 (18.9)	38 (23.2)	43 (34.7)	
ICI modalities					
Nivolumab	263 (38.5)	181 (45.7)	57 (34.8)	25 (20.2)	<0.01
Pembrolizumab	191 (27.9)	125 (31.6)	41 (25.0)	25 (20.2)	
Ipilimumab	81 (11.8)	32 (8.1)	27 (16.5)	22 (17.7)	
Nivolumab + Ipilumumab	71 (10.4)	13 (3.3)	18 (11.0)	40 (32.3)	
Other	78 (11.4)	45 (11.4)	21 (12.8)	12 (9.7)	

Abbreviations: ECOG PS: Eastern Cooperative Oncology Group Performance Status, ICI: immune checkpoint inhibitor, irAEs: immune-related adverse events. * A chi-square test was used to calculate the *p*-value, and Fisher’s exact test was applied if the number in the cell was smaller than 5.

**Table 2:** Association between pre-treatment BMI with irAEs (*N* = 684).

Pre-treatment BMI (kg/m^2^)	Had irAEs/Total	Rate (%) of irAEs and 95% CI	cOR and 95% CI	aOR and 95% CI
<25	115/322	35.7 (30.7, 41.1)	REF	REF
25 to <30	102/217	47.0 (40.0, 53.7)	1.60 (1.12, 2.27)	1.46 (1.02, 2.11)
≥30	71/145	49.0 (40.9, 57.0)	1.73 (1.16, 2.57)	1.47 (0.96, 2.23)
			*p*-trend < 0.01	*p*-trend = 0.04

Abbreviations: aOR: adjusted odds ratio, BMI: body mass index, CI: confidence interval, cOR: crude odds ratio, irAEs: immune-related adverse events. The multivariable model adjusted for age, race, sex, smoking status, metastasis, and lines of therapy.

**Table 3:** Association of pre-treatment BMI (≥25 vs. <25 kg/m^2^) with irAEs in subgroups.

Subgroup	Had irAEs/Total (%)	aOR and 95% CI	*p*-Interaction
Age at first dose of ICI (years)			
<65	147/322 (45.7)	2.18 (1.36, 3.51)	0.02
≥65	141/362 (39.0)	1.08 (0.69, 1.69)	
Sex			
Male	170/394 (43.1)	1.31 (0.84, 2.02)	0.29
Female	118/290 (40.7)	1.73 (1.04, 2.89)	
Race			
White	204/424 (48.1)	1.72 (1.13, 2.61)	0.18
Non-white	84/260 (32.3)	1.16 (0.67, 1.99)	
Multimorbidity			
No	93/213 (43.7)	4.20 (2.11, 8.37)	<0.01
Yes	195/471 (41.4)	1.03 (0.69, 1.52)	
Pre-treatment ECOG			
<2	259/537 (48.2)	1.48 (1.03, 2.13)	0.50
≥2	29/147 (19.7)	1.01 (0.41, 2.48)	
Cancer type			
Lung	85/269 (31.6)	1.34 (0.78, 2.33)	0.99
Melanoma	129/204 (63.2)	1.32 (0.69, 2.51)	
ICI dosage			
1–4	134/372 (36.1)	1.29 (0.81, 2.05)	0.92
≥5	154/312 (49.4)	1.42 (0.87, 2.30)	
ICI type			
Nivolumab ^†^	82/263 (31.2)	0.93 (0.54, 1.60)	0.21
Pembrolizumab ^‡^	66/191 (34.6)	1.92 (1.00, 3.71)	
Ipilumumab ^§^	107/152 (70.4)	1.21 (0.55, 2.65)	

Abbreviations: aOR: adjusted odds ratio, BMI: body mass index, CI: confidence interval, ECOG PS: Eastern Cooperative Oncology Group Performance Status, ICI: immune checkpoint inhibitor, irAEs: immune-related adverse events. The multivariable model was adjusted for the same set of covariates as the primary analysis except the variable used for stratification. ^†^ Among these patients, 133 (50.6%) had lung cancer, 22 (8.4%) had melanoma, and 108 (41.0%) had other types of cancer. ^‡^ Among these patients, 78 (40.8%) had lung cancer, 39 (20.4%) had melanoma, and 74 (38.8%) had other types of cancer. ^§^ Among these patients, 71 (46.7%) used ipilumumab plus nivolumab. A total of 5 (3.3%) patients had lung cancer, 138 (90.8%) had melanoma, and 9 (5.9%) had other types of cancer.

## Data Availability

The datasets used and/or analyzed during the current study are available from the corresponding author on reasonable request.

## Supplementary Materials

The following are available online at https://www.mdpi.com/2072-6694/13/23/6109/s1, Table S1: Summary of each individual irAE. Table S2: Effect measures of other covariates in the primary multivariable logistic regression. Table S3: Effect measures of BMI obtained via multiple imputation. Table S4: Association between pre-treatment BMI and number of irAEs.
