# Correction: Zhang et al. Association between Body Mass Index and Immune-Related Adverse Events (irAEs) among Advanced-Stage Cancer Patients Receiving Immune Checkpoint Inhibitors: A Pan-Cancer Analysis. *Cancers* 2021, *13*, 6109

**DOI:** 10.3390/cancers14184525

**Published:** 2022-09-19

**Authors:** Dongyu Zhang, Neil J. Shah, Michael Cook, Matthew Blackburn, Michael T. Serzan, Shailesh Advani, Arnold L. Potosky, Subha Madhavan, Anas Belouali, Michael B. Atkins, Dejana Braithwaite

**Affiliations:** 1 Department of Epidemiology, College of Public Health and Health Professions, University of Florida, Gainesville, FL 32610, USA; 2 University of Florida Health Cancer Center, Gainesville, FL 32610, USA; 3 Department of Medicine, Solid Tumor Genitourinary Oncology Service, Memorial Sloan Kettering Cancer Center, New York, NY 10065, USA; 4 Department of Medicine, Division of Hematology/Oncology MedStar Georgetown University Hospital, Washington, DC 20007, USA; 5 Department of Oncology, School of Medicine, Georgetown University, Washington, DC 20007, USA; 6 Transplant Education Research Center, Terasaki Institute of Biomedical Innovation, Los Angeles, CA 90024, USA; 7 Georgetown Lombardi Comprehensive Cancer Center, Washington, DC 20057, USA; 8 Innovation Center for Biomedical Informatics, Georgetown University Medical Center, Washington, DC 20007, USA; 9 Department of Surgery, University of Florida, Gainesville, FL 32610, USA

## Addition of Authors

Subha Madhavan (S.M.) and Anas Belouali (A.B.) were not included as authors in the published article [1]. The corrected Author Contributions Statement appears below. Subha Madhavan should be listed as the eighth author, between Arnold L. Potosky and Anas Belouali, and be affiliated with the “Georgetown Lombardi Comprehensive Cancer Center” and “Innovation Center for Biomedical Informatics, Georgetown University Medical Center” (affiliation number 7 and 8). Anas Belouali should be listed as the ninth author, between Subha Madhavan and Michael B. Atkins, and be affiliated with the “Georgetown Lombardi Comprehensive Cancer Center” and “Innovation Center for Biomedical Informatics, Georgetown University Medical Center” (affiliation number 7 and 8).

S.M. and A.B. made substantial contributions to this manuscript. S.M. and A.B. contributed to data curation and cleaning, database maintenance, and editing and review of the manuscript. S.M. also contributed to funding acquisition.

## Additional Affiliation

The affiliation 8 is newly added:

^8^ Innovation Center for Biomedical Informatics, Georgetown University Medical Center, Washington, DC 20007, USA

The affiliation 9 should be updated to:

^9^ Department of Surgery, University of Florida, Gainesville, FL 32610, USA

**Author Contributions:** Conceptualization, D.Z. and D.B.; methodology, D.Z.; software, D.Z.; validation, D.Z., N.J.S., S.A., A.L.P., M.B.A. and D.B.; formal analysis, D.Z.; investigation, D.Z. and N.J.S.; resources, S.M., A.B. and M.B.A.; data curation, S.M., A.B., N.J.S. and M.B.A.; writing—original draft preparation, D.Z.; writing—review and editing, D.Z., N.J.S., M.C., M.B., M.T.S., S.A., A.L.P., S.M., A.B., M.B.A. and D.B.; visualization, D.Z.; supervision, M.B.A. and D.B.; funding acquisition, S.M. and M.B.A. All authors have read and agreed to the published version of the manuscript.

The authors apologize for any inconvenience caused and state that the scientific conclusions are unaffected. The original article has been updated.
