# Supplementary material for: Association between Body Mass Index and Immune-Related Adverse Events (irAEs) among Advanced-Stage Cancer Patients Receiving Immune Checkpoint Inhibitors: A Pan-Cancer Analysis
Source: Cancers (Basel). 2021 Dec 3;13(23):6109. doi: 10.3390/cancers13236109 (PMC8657283; doi:10.3390/cancers13236109)
Supplement: Supplementary file 1 [file cancers-13-06109-s001.zip › cancers-1455217-supplementary.pdf]

cancers

MDPI

Supplementary Materials: Association between Body Mass Index and Immune-Related Adverse Events (irAEs) among Advanced-Stage Cancer Patients Receiving Immune Checkpoint Inhibitors: A Pan-Cancer Analysis

Dongyu Zhang, Neil Shah, Michael Cook, Matthew Blackburn, Michael Serzan, Shailesh Advani, Arnold L. Potosky, Michael B. Atkins and Dejana Braithwaite

Table S1. Summary of each individual irAEs.

	**Overall (*N* = 684) *N* (%)**
Colitis	
No	615 (89.9)
Yes	69 (10.1)
Hepatitis	
No	617 (90.2)
Yes	67 (9.8)
Skin rash	
No	572 (83.6)
Yes	112 (16.4)
Pruritus	
No	650 (95.0)
Yes	34 (5.0)
Other skin toxicities	
No	676 (98.8)
Yes	8 (1.2)
Pneumonitis	
No	654 (95.6)
Yes	30 (4.4)
Hypothyroidism	
No	630 (92.1)
Yes	54 (7.9)
Hyperthyroidism	
No	662 (96.8)
Yes	22 (3.2)
Hypophysitis	
No	664 (97.1)
Yes	20 (2.9)
Other endocrine toxicities	
No	670 (98.0)
Yes	14 (2.0)
Immune-related joint pain or arthritis	
No	670 (98.0)
Yes	14 (2.0)
Immune-related neurological toxicity	
No	675 (98.7)
Yes	9 (1.3)
Immune-related hematological toxicity	
No	678 (99.1)
Yes	6 (0.9)
Musculoskeletal toxicity	
No	674 (98.5)
Yes	10 (1.5)
Other toxicities	

No	653 (95.5)
Yes	31 (4.5)

Abbreviation: irAEs: immune-related adverse events.

Table S2. Effect measures of other covariates in the primary multivariable logistic regression.

Variables	aOR and 95% CI
Age at first dose (years)	
≤54	REF
55-64	0.77 (0.48, 1.23)
65-74	0.66 (0.42, 1.05)
≥75	0.53 (0.32, 0.88)
	p-trend = 0.01
Sex	
Female	REF
Male	1.00 (0.72, 1.38)
Race	
White	REF
Black	0.49 (0.33, 0.73)
Other	0.73 (0.43, 1.21)
Smoking status	
Never	REF
Former	0.88 (0.62, 1.28)
Current	0.86 (0.48, 1.56)
Metastasis	
No	REF
Yes	0.85 (0.56, 1.30)
Line of therapy	
1	REF
2	0.69 (0.49, 0.99)
3+	0.40 (0.25, 0.65)
	p-trend < 0.01

Abbreviations: aOR: adjusted odds ratio, CI: confidence interval.

Table S3. Effect measures of BMI obtained via multiple imputation.

BMI (kg/m^2)	aOR and 95% CI
<25	REF
25-29.9	1.46 (1.04, 2.05)
≥30	1.42 (0.96, 2.09)
	p-trend = 0.04

Abbreviations: aOR: adjusted odds ratio, BMI: body mass index, CI: confidence interval. The logistic regression model adjusted for the same sets of covariates as the primary model and effect measures were estimated by multiple imputation using 5 replicates of chained equations.

Table S4. Association between pre-treatment BMI and number of irAEs.

BMI (kg/m^2)	No. of irAEs and 95% CI	aMD and 95% CI
<25	0.6 (0.5, 0.7)	REF
25-29.9	0.8 (0.7, 1.0)	0.16 (-0.03, 0.34)
≥30	0.9 (0.7, 1.1)	0.20 (-0.02, 0.41)
		p-trend = 0.045

Abbreviations: aMD: adjusted mean difference, BMI: body mass index, CI: confidence interval, irAEs: immune-related adverse events. The multivariable linear model adjusted for the same set of covariates as the primary model.
